# Spatial and temporal influences on discrimination of vibrotactile stimuli on the arm

**DOI:** 10.1007/s00221-019-05564-5

**Published:** 2019-06-07

**Authors:** Valay A. Shah, Maura Casadio, Robert A. Scheidt, Leigh A. Mrotek

**Affiliations:** 1 Department of Biomedical Engineering, Marquette University and Medical College of Wisconsin, Milwaukee, WI USA; 2 DIBRIS, University of Genova, Genova, Italy; 3 Feinberg School of Medicine, Northwestern University, Chicago, IL USA; 4 Division of Civil, Mechanical and Manufacturing Innovation, National Science Foundation, Alexandria, VA USA

**Keywords:** Vibrotactile stimulation, Discrimination threshold, Perception, Dermatomes of the arm, Stimulation timing

## Abstract

Body–machine interfaces (BMIs) provide a non-invasive way to control devices. Vibrotactile stimulation has been used by BMIs to provide performance feedback to the user, thereby reducing visual demands. To advance the goal of developing a compact, multivariate vibrotactile display for BMIs, we performed two psychophysical experiments to determine the acuity of vibrotactile perception across the arm. The first experiment assessed vibration intensity discrimination of sequentially presented stimuli within four dermatomes of the arm (C5, C7, C8, and T1) and on the ulnar head. The second experiment compared vibration intensity discrimination when pairs of vibrotactile stimuli were presented simultaneously vs. sequentially within and across dermatomes. The first experiment found a small but statistically significant difference between dermatomes C7 and T1, but discrimination thresholds at the other three locations did not differ. Thus, while all tested dermatomes of the arm and hand could serve as viable sites of vibrotactile stimulation for a practical BMI, ideal implementations should account for small differences in perceptual acuity across dermatomes. The second experiment found that sequential delivery of vibrotactile stimuli resulted in better intensity discrimination than simultaneous delivery, independent of whether the pairs were located within the same dermatome or across dermatomes. Taken together, our results suggest that the arm may be a viable site to transfer multivariate information via vibrotactile feedback for body–machine interfaces. However, user training may be needed to overcome the perceptual disadvantage of simultaneous vs. sequentially presented stimuli.

## Introduction

Even the simplest actions—such as reaching out toward a coffee mug—typically require the central nervous system (CNS) to integrate information from multiple sensory modalities for planning and executing the motor commands required to accomplish the task [c.f. (Scott 2004)]. In healthy individuals, vision (to locate the desired object relative to the hand) and intrinsic proprioception (to sense body configuration and movement) play key roles in these processes (Sober and Sabes 2003). Unfortunately, diseases such as Parkinson’s Disease (Vaugoyeau et al. 2007), multiple sclerosis (Gandolfi et al. 2015), and neuromotor injury [e.g., spinal cord injury (Crewe and Krause 2009) and stroke (Dukelow et al. 2009)], can interrupt sensory feedback pathways that normally contribute to the accuracy and coordination of movements [c.f., (Sainburg et al. 1993; Sainburg et al. 1995)]. Recent efforts in the development of non-invasive body–machine interfaces (BMIs) have sought to mitigate sensorimotor impairments due to disease and injury using technology to compensate for the sensory and/or motor deficits (Mussa-Ivaldi and Miller 2003).

Various approaches to the development of sensory BMIs have included auditory, haptic, and electro-stimulation [c.f., (Mussa-Ivaldi and Miller 2003; Casadio et al. 2012)]. Vibrotactile feedback (VTF) is an inexpensive and non-invasive way of conveying supplemental information to a user without taxing visual or auditory attention. Common forms of vibrotactile cues include continuous state feedback (Risi et al. 2019; Krueger et al. 2017; Ferris and Sarter 2011), continuous error feedback relative to some goal (Cuppone et al. 2016; Wall et al. 2001; Tzorakoleftherakis et al. 2016), and indicators of undesirable conditions [i.e., alarms; (Ferris and Sarter 2011)]. In each of these cases, the vibrotactile cues should be designed so that the encoded information is clearly perceptible. Moreover, the amount of information that can be encoded by vibrotactile stimuli will depend on the user’s ability to discriminate between different levels of stimulus intensity.

Vibrotactile perception has been studied widely and has advanced the development of technologies for presentation of vibrotactile stimuli [e.g., (Cholewiak 1999; Cholewiak and Collins 2003; Wentink et al. 2011; Verrillo 1985; Harris et al. 2006; Tannan et al. 2007; Ferris and Sarter 2011)]. Perception of vibrotactile stimuli depends on the location of stimulation, inter-stimulus timing, and cognitive ability of the user (Cholewiak 1999; Cholewiak and Collins 2003). Many of these prior studies have focused on the hand and digits as targets of stimulation (Verrillo 1985; Harris et al. 2006; Morley and Rowe 1990; Post et al. 1994; Tannan et al. 2007), because these locations have the highest density of tactile mechanoreceptors (Hunt 1974; Burgess 1973). Because the hand and digits are regularly used for dexterous interaction with the environment, the arm may be a more appropriate site to apply vibrotactile cues. However, few investigations have examined perception and discrimination of vibrotactile stimuli applied to the arm, especially for locations other than the volar forearm.

Our study builds upon prior studies of vibrotactile perception. Mahns et al. (2006) compared vibrotactile frequency discrimination in glabrous versus hairy skin. The discrimination threshold (quantification of discriminability) is defined as the just noticeable difference (JND) between two stimuli. Mahns et al. (2006) reported different discrimination thresholds between the glabrous skin of the fingertip (27.2 Hz) and the hairy skin of the forearm (33.9 Hz), for vibrotactile stimuli frequencies near 200 Hz. Other studies of vibrotactile perception have examined the volar forearm (Post et al. 1994; Cholewiak and Collins 2003; Lamore and Keemink 1988; Morioka et al. 2008), but other locations on the arm have rarely been studied (e.g., medial forearm, dorsal forearm, and upper arm). Furthermore, it is difficult to generalize vibration perception of the hand and digits to that of the arm because the extent to which mechanoreceptor densities differ across the dermatomes of the arm is yet unknown.

Dermatomal representation within primary somatosensory cortex (S1) may also influence our ability to discriminate tactile stimuli. Non-human primate studies have shown that afferent signals from the different dermatomes of the body are projected onto S1 in a way that preserves the arrangement of the spinal segments (Woolsey et al. 1943; Werner and Whitsel 1968). Woolsey et al. (1943) found that cervical dermatomes C2–C8, which span the upper extremity and neck, are projected to large and overlapping areas of S1. By contrast, thoracic dermatomes T1–T12 are mapped onto a single, smaller area. Moreover, there is minimal overlap between the projections of cervical and thoracic dermatomes. This projection pattern may be similar to that in humans (Penfield and Boldrey 1937; Eickhoff et al. 2006). Consistent with this notion, human neuroimaging results show that the proximity of tactile stimulation, both in terms of body part (dermatomal proximity; hemispheric) and in time (i.e., whether the stimuli are presented simultaneously or sequentially), induces different levels of interaction between somatosensory-evoked responses in primary and secondary (S2) somatosensory cortices (Hoechstetter et al. 2001). It is, therefore, possible that systematic variations in neural responses to tactile stimuli separated in space (Duncan and Boynton 2007) and time (Hoechstetter et al. 2001) may influence our ability to discriminate vibrotactile stimuli applied to different dermatomes in the arm and hand. In the present study, we sought to test this hypothesis by quantifying the ability of human subjects to discriminate pairs of vibrotactile stimuli of differing intensities when applied simultaneously and sequentially to various locations on the arm.

Perceptual decision-making involves several central processes (including memory and attention) that contribute to the comparison of sensory stimuli (Heekeren et al. 2008). Discriminating between two sequential stimuli requires a neural representation of the first stimulus to be stored in working memory, which can later be accessed to compare against a second stimulus (Romo et al. 2002). Stimuli stored as neural responses are subject to noise and fading (forgetting), both of which can degrade the response and lead to worse discriminability [c.f. (Bernasconi et al. 2011; Harris et al. 2002)]. Focusing attention towards a sensory stimulus allows for less neuronal response variability (Mitchell et al. 2007). For accurate perception in the case of simultaneous stimuli, attentional resources must be divided between the two stimuli (Connell and Lynott 2012). Dividing attention across multiple sensory inputs increases neuronal variability (Mitchell et al. 2007) and introduces information leakage (from unimportant sensory stimuli) that can bias the decision-making process (Wyart et al. 2015). Thus, discrimination of two vibrotactile stimuli presented in different locations is influenced not only by the stimulation sites, but also by the relative timing of the stimuli (i.e., whether they are delivered sequentially or simultaneously).

In this study, we sought to describe how spatial and temporal features of vibrotactile stimuli influence their perception. Using an experimental setup wherein the amplitude and frequency of vibration covary, we performed a series of two-alternative forced-choice experiments that quantified discrimination of sequential and simultaneous vibrotactile stimulus intensities within and across dermatomes of the arm and hand. The experiments were designed to test two hypotheses. First, based on differences in mechanoreceptor density and cortical representation across dermatomes, we hypothesized that the acuity of vibration intensity discrimination differs across dermatomes of the arm. Second, based on the contributions of attention and working memory on perceptual decision-making, we hypothesized that discrimination of vibrotactile stimuli is additionally influenced by inter-stimulus timing (i.e., sequential vs. simultaneous presentation). We analyzed the JNDs of vibrotactile stimulus intensities to determine the effects of stimulus location and inter-stimulus timing on the perception of vibrotactile stimuli. We expect that our results will enhance the utility of vibrotactile feedback in applications such as grip force feedback in the control of prosthetic hands (An et al. 2011), kinesthetic feedback for limb movement control in survivors of stroke (Krueger et al. 2017), and offloading of visual attention in spinal cord injury patients learning a brain–machine interface (Cincotti et al. 2007).

## Materials and methods

### Participants

Thirty neurologically intact participants (14 females; 16 males) with no known cognitive deficits or tactile deficits of the arm were recruited from the Marquette University community. Participants ranged in age from 19 to 29 years (22.9 ± 2.05 yrs, mean ± SD; there was no significant age difference between the male and female subsets). Participants gave written, informed consent to participate in one of two experiments. All experimental procedures were approved by Marquette University’s Institutional Review Board in full accordance with the Declaration of Helsinki.

### General Experimental Setup

**Fig. 1:**
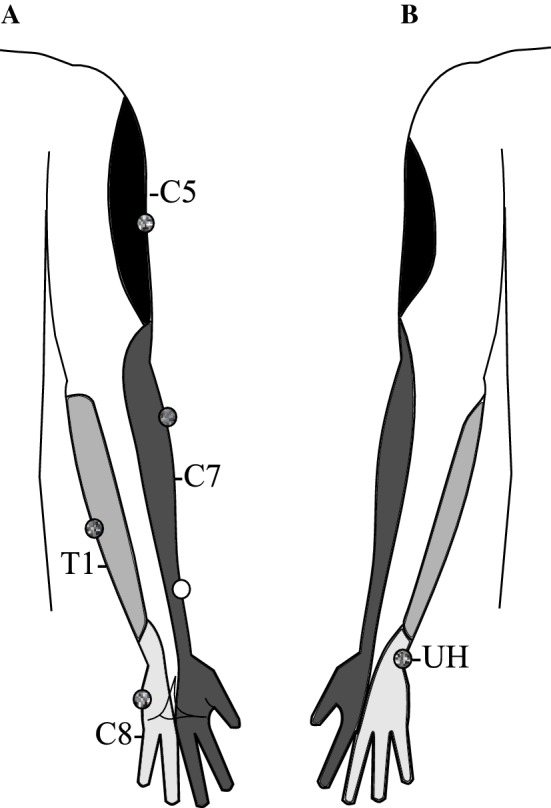
Mechanoreceptors within the arm and hand send afferent projections to one or more segments of the spinal cord through the Dorsal Root Ganglia. The dermatomes of the arm (the domains of origin of those projections) are labeled according to their target cord segment, and are marked by the *shaded regions*. The *white shaded regions* are areas of major dermatomal overlap, i.e., more than 1 spinal cord segment can innervate that region. **a** The anterior view of the arm, showing dermatomes, C5, C7, C8, and T1. **b** The posterior view of the arm, showing dermatomes and the Ulnar Head. The *gray markers* indicate the placement of the vibration motor motors on the arm in experimental 1 and 2. The *white marker* indicates the placement of the second vibration motor during the C7–C7 pair of experimental 2. Adapted from Lee et al. (2008)

Participants were seated with their dominant arm (self-reported) supported by a 1-inch-thick memory foam pad on top of a table. The elbow was oriented at 90° relative to the torso, with approximately 15° of shoulder flexion, and no shoulder ab/adduction. The forearm was relaxed on the foam pad with the lateral forearm supinated, such that the palm faced upward. Vibrotactile stimuli were delivered to the arm and hand via 10 mm eccentric rotating mass (ERM) vibration motors (Precision Microdrives Ltd, Model # 310–117) with an operational frequency range of approximately 60–240 Hz, which corresponded to an amplitude range of 0.5–2.4 G. For simplicity, we chose to represent vibrotactile stimulus intensity in terms of frequency even though the amplitude of vibration covaried with frequency in the ERM vibration motors [c.f. Hwang et al. (2013) for a description of how perception of vibration intensity changes as vibration frequency and amplitude change]. The vibration motors were powered and controlled using drive circuitry that was interfaced to a portable laptop computer running a custom script within the MATLAB R2017a computing environment (MathWorks Inc., Natick MA). Vibration motors could be placed on five locations: dermatome C5, C7, C8, T1, or the ulnar head (UH), a boney prominence within the projection of dermatome C8. Figure 1 shows the dermatomes of the arm and the approximate locations of the testing sites. Vibration motors were fixed to the arm via Transpore tape (3M Inc).

### Constant stimuli protocol

We conducted a series of two-alternative forced-choice experiments (2-AFC) using the method of constant stimuli (Gescheider 1997) to determine the JND of vibrotactile stimulus intensity for each participant under various testing conditions. The 2-AFC protocol presented participants with a series of 110 stimulus pairs, each comprised of a standard intensity that remained fixed throughout the experimental session, and a probe intensity that varied across stimulus pairs. The standard intensity for our experiments was set to a frequency (186 Hz), approximately in the middle range of the Pacinian Corpuscle’s frequency sensitivity band [60–400 Hz; (Mountcastle et al. 1972); see also (Ribot-Ciscar et al. 1989)]. The probe intensity included five intensities below the standard, five intensities above the standard (ranging from 100 to 235 Hz; corresponding amplitude of 0.45–2.25 G), and the standard intensity itself (186 Hz; corresponding amplitude of 1.40 G).

For experiment 1, a single vibration motor was used to present two sequential vibrations at each one of five different locations. We asked participants to verbally indicate which stimulus, first or second, was perceived to be of greater intensity. For experiment 2, two vibration motors were used to present pairs of vibrations (sequentially or simultaneously) across pairs of stimulation sites. In this case, we asked participants to verbally indicate the location of the stimulus perceived to be of greater intensity.

### Presentation of stimuli

During the *sequential* presentation of stimuli, the first vibrotactile stimulus was delivered for 750 ms, followed by a 750 ms pause, and then, the second stimulus was presented for 750 ms. During the *simultaneous* presentation of stimuli, both vibrotactile stimuli were presented at the same time for duration of 750 ms. This presentation method was only used for experiment 2, wherein two vibration motors delivered vibrotactile stimuli to several location pairs.

### Experiment 1: discrimination thresholds for sequential stimuli applied at single locations in dermatomes of the arm and hand

Fifteen participants (6 females) volunteered to participate in three experimental sessions, lasting approximately 60 min each, spaced at least 24 h apart. Each session consisted of five blocks of 2-AFC trials. During each block, one vibration motor was attached to the arm at one of five arm locations: C5, C7, C8, T1, or UH (Fig. 1: *gray markers*). The vibrotactile discrimination threshold was tested using sequential stimuli presentation as described in *Constant Stimuli Protocol* above. Participants completed 110 trials during each block (11 probe stimuli repeated 10 times each), wherein they verbally indicated which of the two stimuli they perceived to be more “intense”, regardless of whether they interpreted stimulus intensity to refer to stimulus amplitude or frequency (which were coupled by the ERM motors used in these experiments). Each trial lasted about 2–4 s depending on participant response time; between each trial, there was a 2–3 s rest period. The ordering of standard and probe stimuli presentation (i.e., which stimulus was presented first) was pseudorandomized across trials. Testing locations were also pseudorandomized across participants and sessions to minimize potential order effects.

### Experiment 2: sequential versus simultaneous stimulations within and across dermatomes

Fifteen participants (8 females) volunteered to participate in a single experimental session lasting approximately 90 min. The session consisted of eight blocks of 2-AFC trials. During each block, one of four dermatomal pairs was tested using either sequential or simultaneous presentations: within a dermatome (C7–C7) and across dermatomes (C7–C5, C7–UH, and C7–T1). One vibration motor was always placed on dermatome C7 at the location marked by the *gray* C7 marker in  Fig. 1. A second vibration motor was attached to the other indicated location. We performed a pilot study that used a vibration motor and a three-axis accelerometer to measure the propagation of vibrations across the arm; we found that interference across stimulation sites was negligible with motor separations greater than 8 cm [data not shown; see also (Krueger et al. 2017; Cipriani e al. 2012)]. The two vibration motors were, therefore, always placed at least 8 cm apart.

The vibrotactile discrimination threshold was tested using sequential or simultaneous stimuli presentation as described in *Constant Stimuli Protocol* above. Participants completed 110 trials during each block, where they verbally indicated which of the two tested locations received the more “intense” stimulation. The ordering of standard and probe stimuli (i.e., which stimulus was presented at which location) was pseudorandomized across trials. Each trial lasted about 2–4 s depending on participant response time, and between each trial, there was a 2–3 s rest period. Block presentation order [i.e., the eight combinations of stimulation delivery method (sequential/simultaneous) and sites (dermatomal pairs)] were also pseudorandomized across participants and blocks to minimize potential order effects.

### Data analysis

Verbal responses were converted into probabilities of indicating each probe intensity as greater than the standard intensity. For each participant and each testing block, psychometric functions were fitted to the probability data as a function of probe stimulus intensity (represented by frequency) using the cumulative normal distribution (Eq. 1):

1 $$F(x) = \frac{1}{2}\left[ {1 + {\text{erf}}\left( {\frac{x - \mu }{\sigma \sqrt 2 }} \right)} \right],$$

where *F*(*x*) is the predicted probability, *x* is the probe intensity, *μ* is the mean of the underlying decision process modeled as a normal distribution, σ is the standard deviation of that normal distribution, and the erf is the cumulative normal function. Curve fitting was performed using the MATLAB function (*fminsearch*) to find the *μ* and σ values that minimized the sum of squared error between the predicted and actual response probabilities. The vibrotactile intensity discrimination threshold was defined as one standard deviation of the underlying normal distribution (i.e., the σ found by *fminsearch*). This discrimination threshold (i.e., the JND) was defined as a measure of uncertainty in comparing vibration intensities near the standard intensity of 186 Hz. For probe stimuli either much greater than or much less than the standard stimulus, we expect people to be relatively accurate in discriminating the probe and standard stimulus intensities. As we found no significant effect of sessions for experiment 1 (see “Results”), discrimination thresholds were averaged across the three sessions for each tested location, to yield one discrimination threshold per participant per condition. For both experiments 1 and 2, we report the mean discrimination threshold averaged across participants and within blocks.

### Statistical hypothesis testing

Motivated by the observation that the density of cutaneous mechanoreceptors varies across the body (Hunt 1974), we first sought to test the extent to which discrimination thresholds for vibrotactile stimuli might vary across locations of the arm and hand (Experiment 1). Specifically, we used two-way ANOVA and post hoc, Bonferroni-corrected, paired samples *t* test to compare mean vibrotactile discrimination thresholds (the dependent variable) across sessions and across locations on the arm and hand.

Motivated by the consideration that discrimination of sequential vibrotactile stimuli involves aspects of working memory and attention, which might be limited resources and divided for simultaneously presented stimuli, we sought to test the hypothesis that discrimination thresholds would vary between sequential and simultaneously presented stimuli, both within and across dermatomes (Experiment 2). We used two-way ANOVA and post hoc, Bonferroni-corrected, paired samples *t* test to compare mean discrimination thresholds (the dependent variable) across delivery methods (sequential or simultaneous) and across location of stimulus delivery (within or across dermatomes).

All analyses were performed with SPSS Statistics 24 (IBM Corp, Armonk, NY). Statistical significance was set at the family wise error rate of *α* = 0.05.

## Results

This study used eccentric rotating mass (ERM) vibration motors to examine the psychophysics of vibrotactile perception within and across dermatomes of the arm and hand in 30 neurologically healthy participants. All participants were attentive throughout their experimental session, and all responded to stimuli in a timely fashion.

### Experiment 1: discrimination thresholds for sequential stimuli applied at single locations in dermatomes of the arm and hand

**Fig. 2:**
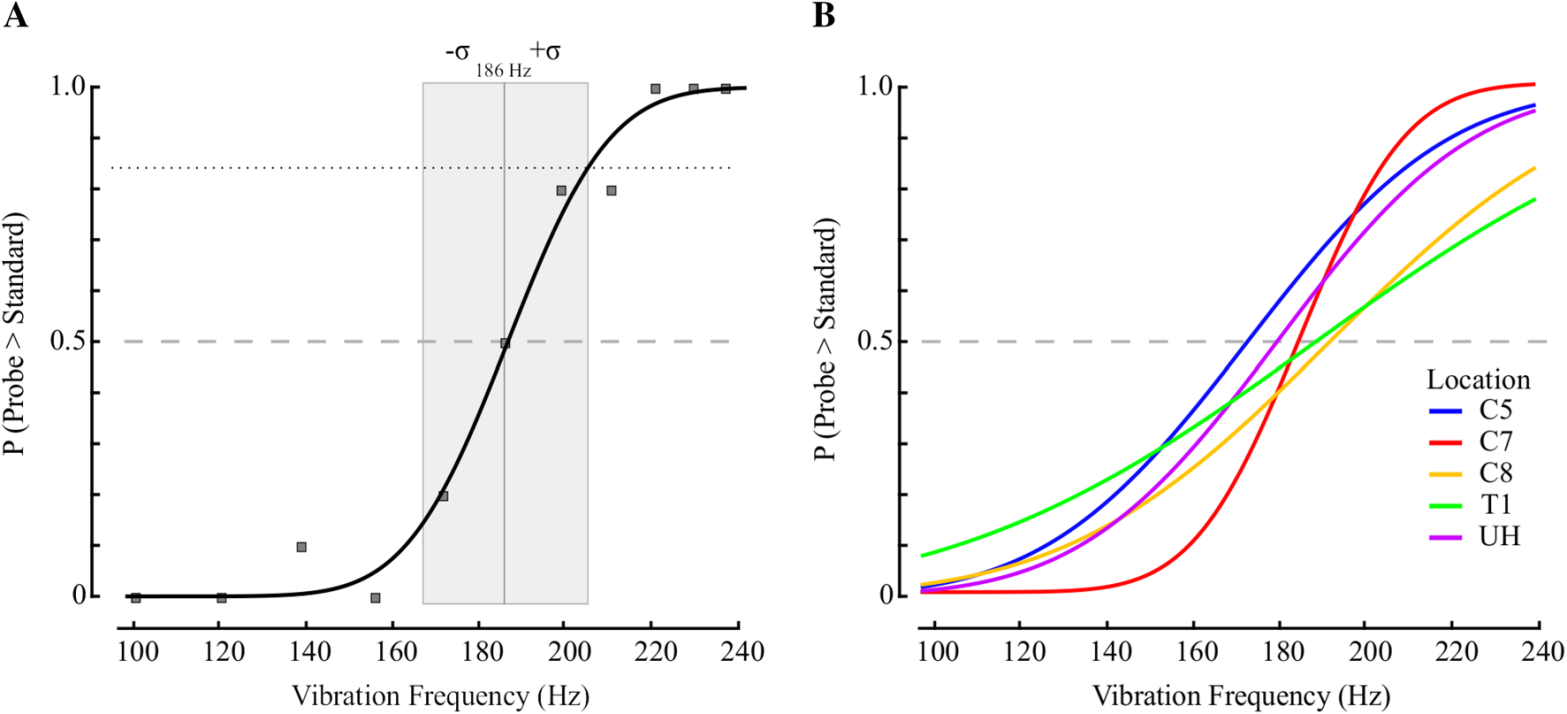
**a** Assessment of vibrotactile perception at dermatome C7 for a selected participant. *Gray squares* indicate the observed fraction of trials at each probe frequency where the participant indicated that they perceived the probe stimulus as more intense than the standard stimulus. *Black sigmoid curve*: the psychometric (cumulative normal) function that was fit to the observed probability data. *Gray shaded region*: the discrimination threshold defined as one estimated standard deviation (here, ± 19.01 Hz) from the estimated mean (186.38 Hz) of the underlying normal distribution. The upper bound of the box crosses the sigmoid at approximately *P(Probe > Standard)* = 0.84 (*gray dotted line*). *Gray dashed line*: the point of subjective equality (i.e., *P(Probe >Standard) * = 0.5). **b** Best-fit cumulative normal functions for the five testing locations for the same participant. Dermatome C7 has the best discrimination threshold, while dermatome T1 has the worst

In the first set of experiments, we tested the extent to which difference thresholds for vibrotactile intensity vary across dermatomes of the arm and hand. Figure 2a depicts response probabilities calculated from a single block of discrimination trials performed by one participant (dermatome C7). As expected, when the probe intensity was markedly lower than that of the standard, the participant reliably identified the standard as more intense than the probe [i.e., *P* (probe > standard) was close to 0]. By contrast, when the probe intensity was markedly higher than that of the standard, the participant was much more likely to identify the probe as more intense. When the probe intensity was close to that of the standard, the participant was less reliable in correctly identifying which stimulus was more intense. We fit the cumulative normal function (Eq. 1) to the observed likelihood data to obtain estimates of *µ* and σ from the underlying normal model of the perceptual decision process. Figure 2b presents the psychometric curves obtained from all five testing locations from the same participant. Dermatome C5 is traced by the blue curve (174.27 ± 35.87 Hz; *µ* ± σ of the underlying normal distribution), dermatome C7 by the red curve (186.38 ± 19.01 Hz), dermatome C8 by the orange curve (193.09 ± 46.69 Hz), dermatome T1 by the green curve (189.29 ± 64.42 Hz), and the ulnar head by the purple curve (181.16 ± 34.95 Hz). Here, the psychometric curve for dermatome C7 had the steepest slope (smallest σ), whereas the psychometric curve for dermatome T1 had the shallowest slope (greatest σ). Thus, this participant was better at discriminating between vibrotactile stimuli intensity presented sequentially on dermatome C7 than the same stimuli presented on dermatome T1. Discrimination thresholds for sequential stimuli applied to dermatomes C5 and C8, and the ulnar head fell between the bounds established by dermatomes C7 and T1.

**Fig. 3:**
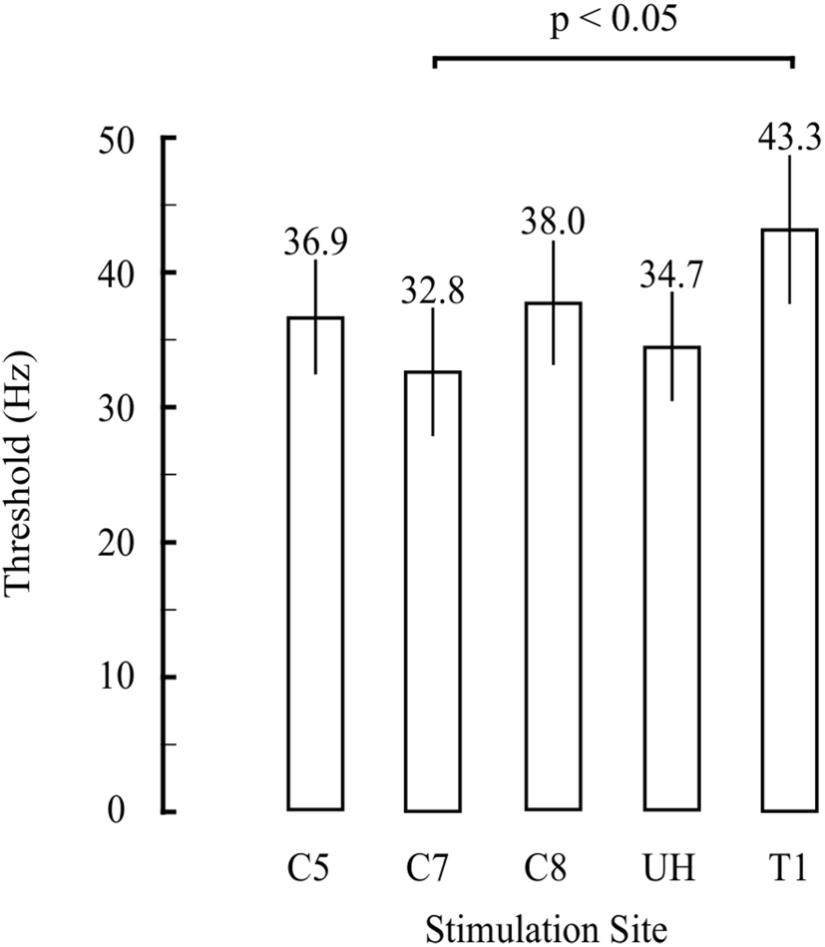
Group results from Experiment 1. Mean (± 1 SEM) discrimination thresholds across the population were calculated for sequential vibrotactile stimuli presented within each of the five tested locations. Dermatome C7 is significantly better at discriminating vibrotactile stimuli than dermatome T1

The results presented in Fig. 2 were representative of the study population (Fig. 3). Two-way ANOVA found that vibrotactile discrimination thresholds differed significantly across stimulation sites (*F*_4,56_ = 6.801, *p* = 0.0002), but not across session (*F*_2,28_ = 1.212, *p* = 0.313). Post hoc testing revealed that this effect was due to better vibrotactile discrimination on dermatome C7 [32.78 ± 4.73 Hz (mean ± SEM)] vs. dermatome T1 (43.25 ± 5.48 Hz, *t*_14_ = 5.22, *p* = 0.0001). Vibrotactile discrimination thresholds on dermatomes C5 (36.88 ± 4.23 Hz), C8 (37.96 ± 4.58 Hz), and the Ulnar Head (34.70 ± 4.03 Hz) did not differ significantly from each other or from those of dermatomes C7 or T1 (*p* > 0.05 in all cases). Across participants, the average difference in discrimination thresholds between dermatomes C7 and T1 was 10.47 ± 1.48 Hz. We also calculated the average slopes of the psychometric functions at its inflection point within each of the tested dermatomes (Slopes: C5 = 0.0159 ± 0.0032 (mean ± SEM), C7 = 0.0240 ± 0.0057, C8 = 0.0182 ± 0.0055, UH = 0.0234 ± 0.0058, T1 = 0.0125 ± 0.0016). It can be shown by differentiating Eq. 1 with respect to *x* that the slope of the psychometric function at the inflection point (i.e., when *x* = *μ*) is a reciprocal function of the discrimination threshold σ. Despite this nonlinearity, the slopes of the fitted psychometric functions exhibited a high degree of negative correlation with discrimination thresholds over the range of the experimentally observed thresholds (*r* = − 0.926).

### Experiment 2: sequential versus simultaneous stimulations within and across dermatomes

**Fig. 4:**
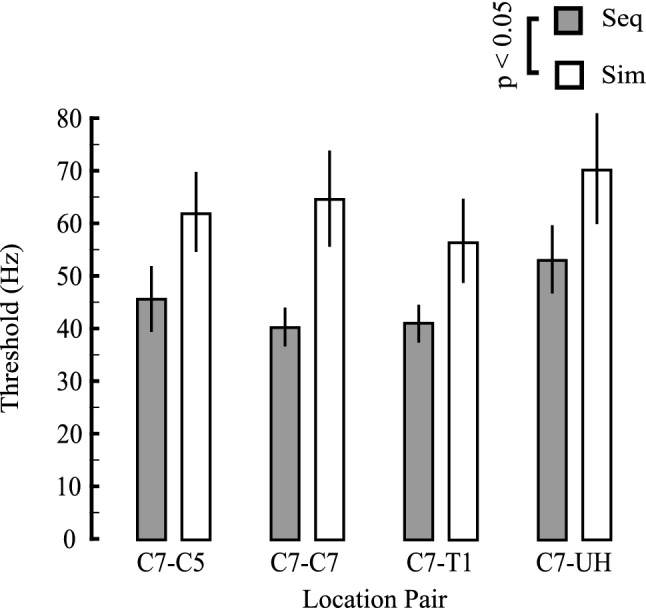
Group results from Experiment 2. Mean (± 1 SEM) discrimination thresholds were calculated for sequentially (*gray bars*) and simultaneously delivered (*white bars*) vibrotactile stimuli at each stimulus location pair. Sequential vibrotactile stimuli (C7–C5: 46.32 ± 6.29 Hz; C7–C7: 40.94 ± 3.70 Hz; C7–T1: 41.74 ± 3.60 Hz; C7–UH: 53.75 ± 6.51 Hz) allowed for better discriminability than simultaneous stimuli (C7–C5: 62.63 ± 7.62 Hz; C7–C7: 65.38 ± 9.17 Hz; C7–T1: 57.06 ± 8.04 Hz; C7–UH: 70.96 ± 10.56 Hz)

In the second set of experiments, we examined two factors having the potential to impact how the CNS processes vibrotactile information in support of perceptual decision-making: concurrency of stimuli (i.e., whether working memory and attention are required to support the decision) and somatotopy of stimulus delivery (i.e., whether the two stimuli are provided within the same dermatome or across different dermatomes). Participants performed eight blocks of 2-AFC trials wherein they discriminated between two vibrotactile stimuli delivered either sequentially or simultaneously at each of four location pairs on the arm or hand; each permutation of this 2 × 4 experimental design was tested in separate blocks. As per Experiment 1, we fitted Eq. 1 to the observed response likelihood data from each block to obtain separate estimates of the mean (*µ*) and standard deviation (*σ*) of the normal model of the perceptual decision process underlying each testing condition. Two-way ANOVA found that vibrotactile discrimination thresholds varied systematically by delivery method (*F*_1, 113_ = 13.01, *p* = 0.0004), but did not vary significantly across paired stimulation sites (*F*_3, 113_ = 1.124, *p* = 0.343). Participants demonstrated better discriminability of vibrotactile stimuli with sequential delivery [45.57 ± 3.92 Hz (mean ± SEM)] than with simultaneous delivery (64.14 ± 6.54 Hz) (Fig. 4). Across participants, the difference in discrimination thresholds between delivery methods averaged 18.57 ± 7.83 Hz. The main effect found in experiment 1 did not differ significantly from the main effect found in experiment 2 (two-sample t test, *t*_28_ = 1.0167, *p* = 0.318).

## Discussion

This study investigated vibration intensity discrimination when stimuli were applied either sequentially or simultaneously to various dermatomes on the arm and hand (C5, C7, C8, and T1). Based on the reports of differing densities of mechanoreceptors in the hand and varying dermatomal representations in the primary (S1) and secondary somatosensory cortex (S2), we hypothesized that the discrimination threshold for vibrotactile stimuli would vary across dermatomes. In support of this hypothesis, we observed that vibrotactile intensity discrimination threshold in dermatome C7 was on average approximately 10 Hz lower than the threshold for dermatome T1. However, the dermatomal effect is only a small fraction of the JND for each dermatome (ranging from 23% in dermatome T1 to 31% in dermatome C7). Thus, this fractional difference is well below the perceptible change in vibration intensity. The current study also tested the hypothesis that discrimination thresholds of vibrotactile stimuli depend on whether the stimuli are delivered sequentially or simultaneously. Our results showed that the discriminability of sequentially delivered stimuli was better than that of simultaneously delivered stimuli. We conclude, therefore, that while all of the tested dermatomes on the arm and hand could serve as viable sites of vibrotactile stimulation for a practical BMI, implementations should ideally account for small differences in perceptual acuity across dermatomes. Moreover, the maximum amount of information that can effectively be encoded will be constrained by at least two factors: limitations in vibrotactile perceptual acuity that differ slightly between dermatomes, and limitations in the amount of information that can be simultaneously presented across multiple stimulation sites.

### Discrimination across dermatomes—possible mechanisms

It is possible that the difference in discrimination thresholds between dermatome C7 and T1 are attributable to differences in the cortical representation of dermatomal projections onto the somatosensory cortex (i.e., the number of neurons responsible for sensing a stimulus). In non-human primates, the cortical representation area is much larger for dermatome C7 than T1 (Woolsey et al. 1943). Dermatomal representations in the somatosensory cortex of the human brain likely follow a similar pattern (Penfield and Boldrey 1937; Eickhoff et al. 2006), suggesting a possible mechanism for the different discrimination levels which we found for dermatomes C7 and T1 in experiment 1. Duncan and Boynton (2007) showed that, in humans, the extent of cortical representation of the index finger is much larger than that of the little finger, and that the cortical representation correlates with tactile acuity in the two fingers. In our study, discrimination thresholds in the cervical dermatomes were indistinguishable, whereas dermatomes C7 and T1 differed significantly in a way that could reflect greater cortical representation of the cervical dermatomes. Future neuroimaging work is needed to test whether cortical representation can explain the differences in discrimination observed in this study.

A second possibility relates to potential differences in mechanoreceptor density across the arm. Pacinian corpuscles (PCs) are much sparser and their location is also much deeper in the epidermis of hairy skin relative to glabrous skin (Burgess 1973). Johansson and Vallbo (1979) showed that the density of PCs is higher towards the lateral side (index finger and thumb) of the hand compared to the medial side (little finger). This lateral-to-medial difference in mechanoreceptor density may also hold true for the forearm. Desensitization of dermatome T1 (medial arm) may also occur due to frequent interactions with objects in the environment (e.g., resting the arm on a chair or a table). To our knowledge, no studies to date have compared mechanoreceptor density or sensitivity across the dermatomes of the arm or other body locations, which could provide valuable insights into differences in discrimination acuity across the dermatomes of the body.

### Discrimination across time: influence of working memory and attention

A comparison of two studies from Romo and colleagues provides insight into the neural correlates of vibrotactile stimulus discrimination when two stimuli are presented sequentially, as in the present study. In a first study, Romo et al. (1999) recorded from neurons in the prefrontal cortex (PFC) of non-human primates. Here, activations reflected the contribution of working memory to the discrimination of two sequential vibrotactile stimuli. During the delay period between the two stimuli, neuronal responses to the first stimulus were maintained within the PFC throughout the delay period. Moreover, the neuronal responses in the PFC within the last 200 ms of the delay period persisted at levels consistent with neuronal responses recorded in the primary (S1) and secondary (S2) somatosensory cortices during the first stimulus. By contrast, little-to-no delay period activations were observed in either S1 or S2 in their later study (Romo et al. 2002). Whereas neuronal responses to the first stimulus depended only on its frequency of vibration in both S1 and S2, neuronal responses to the second stimulus were proportional to the difference in the vibration frequency of the two stimuli (f2–f1) in about 20% of the recorded S2 neurons (but not in S1). Within this subset, Romo and colleagues (2002), through the analysis of trials wherein the monkeys made erroneous choices, found that neuronal responses reflected the actual choice the monkey would ultimately make rather than strictly adhering to the (f2–f1) relationship (see their Fig. 7a). This was true for responses recorded even within the first 300 ms of the second stimulus, well before the motor response to the decision was performed. If the mechanism of stimulus encoding, recall, and discrimination described by Romo and colleagues also holds true for vibrotactile discrimination in humans, then the decreased acuity that we observed during the discrimination of simultaneous stimuli may be due to timing constraints that preclude the engagement of working memory systems located within PFC [(Braver et al. 1997; Lara and Wallis 2015); for review, see (Curtis and D’Esposito 2003)].

Wu and Liu (2008) have compared the structure of information processing within the CNS to computer networking structures, where regions such as the PFC, S1, and S2 act as servers that are connected to each other through routers (neural pathways). In this queuing-network model, Wu and Liu conceptualized that sensory information is processed and routed through multiple servers that comprise different perceptual, cognitive, and motor subnetworks. Whereas simultaneous sensory stimuli can be perceived and stored at the same time in the perceptual subnetwork, one stimulus must be processed before the second within the cognitive subnetwork, because each stimulus must pass serially through the same server. While the memory of one stimulus is waiting to be processed by the cognitive network, noise in the form of neuronal response variability can degrade the stored representation (Bernasconi et al. 2011). By contrast, each of two sequential stimuli can be processed immediately by the cognitive subnetwork if the time between two stimuli exceeds some minimum time required to process a single stimulus. In our study, the inter-stimulus interval of 750 ms evidently exceeded that minimum, because the acuity of vibrotactile discrimination was systematically lower for sequential vs. simultaneous stimuli. A future study of vibrotactile discrimination should manipulate the duration of the inter-stimulus interval to identify the time-course and effects of memory encoding, recall, and forgetting on vibrotactile perceptual acuity [see e.g., (Harris et al. 2002; Berglund et al. 1967; Gallace et al. 2008)].

Variations in attention also likely impact the acuity of vibration intensity discrimination. Attentional resources available for the comparison of vibrotactile stimuli likely follow the capacity sharing model proposed by Pashler (1994). In that model, attention is a limited capacity resource. Attentional capacity that is shared or divided across multiple stimuli reduces the capacity available for perception of each individual stimulus. When attention towards a stimulus decreases, higher variability in neuronal responses can increase neuronal noise (Mitchell et al. 2007). Noise in the representation of a vibrotactile stimulus can also increase due to leakage of information from the other sensory modalities (e.g., audition, vision) that may or may not provide a signal consistent with the vibrotactile stimulus (Mozolic et al. 2008; Wyart et al. 2015). Signal detection theory predicts that the accuracy of discrimination will be degraded by the presence of noise, whatever its source (Green and Swets 1966; Wickens et al. 2015). Attention can act as a filter during the perception of stimuli by attenuating noise (Mozolic et al. 2011), thereby reducing variability in the neuronal response (Mitchell et al. 2007; Bernasconi et al. 2011). Thus, division of attention may have contributed to the systemic increase in discrimination thresholds observed during simultaneous presentation of vibrotactile stimuli in experiment 2.

### Implications for vibrotactile sensory augmentation

By developing an understanding of vibrotactile perception, vibrotactile feedback (VTF) can be used more effectively in applications such as sensory augmentation (Bach-y-Rita 1967; Shull and Damian 2015; Witteveen et al. 2015; Cuppone et al. 2016; Risi et al. 2019). Sensory augmentation is a technique where one sensory modality is enhanced or replaced through the application of stimuli to another sensory modality. The use of vibrotactile feedback in sensory augmentation has been investigated since the 1960s. Previous studies have utilized the tactile sense to augment several other senses. For example, Witteveen et al. (2015) demonstrated that it is possible to improve the control of grip force and hand aperture in prosthetic users by providing feedback of these variables via vibrotactile cues. Cuppone et al. (2016) enhanced performance of wrist movements by supplementing proprioceptive training with error-based vibrotactile feedback provided on either forearm. In our earlier works (Krueger et al. 2017; Risi et al. 2019), we also investigated the use of vibrotactile sensory augmentation for upper extremity motor control. We encoded limb state or performance error information about the moving arm within vibrotactile feedback applied to the other (non-moving) arm. With both forms of information encoding, the use of vibrotactile feedback led to significant improvements in the performance of reaching and stabilization behaviors.

One reason for choosing the arm as a location for vibrotactile feedback is allowing the user to manipulate objects with both hands (e.g., using the non-dominant hand to hold a bottle, while the dominant hand opens it) without obstructing the hand and digits with the vibration motors. Another factor to consider when choosing a location for vibrotactile stimulation is the ease of interpretation of the stimuli. All previous studies involving vibrotactile feedback have selected sites that are in some sense intuitive or relevant to the specific application under examination. For example, we have previously shown the intuitiveness of using vibrotactile feedback applied to the arm to successfully guide reaching (Risi et al. 2019; Krueger et al. 2017). Wall et al. (2001) demonstrated a reduction in body sway during quiet standing in healthy users who were provided vibrotactile feedback to the trunk. In that case, the stimuli conveyed information about head tilt. Sienko et al. (2008) expanded that work by providing vibrotactile error feedback of trunk sway to users with vestibular sensation loss. Doing so successfully reduced body sway. Our current study advances the development of sensory augmentation applications by providing a better understanding of vibration intensity perception on various locations of the arm. The methods described in this study could be used in the future to quantify vibrotactile perception at other body locations suitable for other potential applications (e.g., providing feedback of ankle angle on the thigh to mitigate foot drop).

The current results also provide insight into the maximum amount of information that can be encoded by VTF-based BMIs. The results of our first experiment characterized the minimum intensity difference between two vibrotactile stimuli required to accurately distinguish between them. Given that the bandwidth of human vibration perception via PCs is limited (i.e., 60–400 Hz), the number of discretely perceptible stimuli within that range is determined by the smallest resolvable difference between two stimuli in that range (i.e., the JND). Thus, while all of the tested dermatomes on the arm and hand could serve as viable sites of vibrotactile stimulation for a practical BMI, future applications of vibrotactile sensory augmentation on the arm may consider using dermatomes C5, C7, or C8 (UH) as stimulation sites, because they have indistinguishable discrimination thresholds, while potentially avoiding dermatome T1, which has a slightly elevated discrimination threshold. The results of our second experiment showed that sequential delivery outperforms simultaneous delivery. The implication is that the number of independent vibrotactile channels that can be used to simultaneously convey useful information may be limited, at least upon the initial exposure in untrained individuals, as tested here. Future applications using multichannel vibrotactile stimulation may consider limiting the extent to which attention must be divided across multiple simultaneous stimuli either through the minimization of distractions, or through the promotion of autonomous sensory integration via long-term training.

Finally, the tactile sensory modality also plays a role in body representation and influences proprioception (Weerakkody et al. 2007; Kuling et al. 2016; Lee et al. 2013). Weerakkody et al. (2007) showed that stimulating the cutaneous mechanoreceptor through vibrotactile stimuli decreased perception of proprioceptive changes, leading to decreased detection of movements. The work of Weerakkody and colleagues focused on detection of movements in the digits of the hand, while this same area was also stimulated with vibration; how their findings may generalize to hairy skin of the body is yet unknown. To provide the best utility and experience for the user of novel technology that employs supplemental vibrotactile stimuli, it is important to consider where on the body the cues are to be applied, what information they will provide, and whether the cueing may interfere with other intrinsic modes of somatosensation (e.g., proprioception).

### Limitations

There are several potential limitations of the present study. One limitation might arise from differences in contact force/pressure between vibration motors attached to different stimulation sites. We mitigated this concern by having the same experimenter attach the motors to the skin using medical grade tape, taking care to ensure that the length of tape (~ 4 cm) and tension were consistent across testing sites and participants. We also counter-balanced the presentation of standard and probe stimuli across the two locations through pseudo-randomization to reduce any systematic effects of differences in contact force/pressure.

Another limitation may arise from our use of inexpensive ERM vibration motors rather than more expensive devices that can decouple the frequency of vibration from its amplitude. While the selection of vibrating actuators might affect perception of vibration [c.f. (Lee et al. 2013)], it is unlikely that the factors contributing to the spatiotemporal variations in vibrotactile acuity described in this study would be the result of variations in sensitivity to just one of these parameters (frequency, amplitude) but not the other, and so the overall pattern of results which we describe should not depend on the choice of vibration motor technology. In addition, studies by Choi and Kuchenbecker (2013), Hwang et al. (2013), and Morley and Rowe (1990) have shown that perception of vibration intensity depends both on the frequency and amplitude of vibration. Counterintuitively, Hwang et al. (2013) showed that, at certain frequencies of stimulation, the perceived intensity of vibration can decrease even as the amplitude of vibration increases. Thus, the coupling of vibration magnitude and frequency is a beneficial feature of the low-cost ERM motors in our study. Indeed, as exemplified by the data provided in Fig. 2, the perceived intensity of vibration increased monotonically as a function of motor activation in all subjects in the current study over the range of frequencies stimulated by the selected ERM motors. Therefore, the low-cost ERM vibration motors are well suited for use in VTF applications.

Other limitations might arise from our choices to include only healthy, young participants in this study, to test using only a single standard stimulus, and to test using only a single-stimulus duration. Aging has been shown to be a factor in perception of vibrotactile stimulations (Lin et al. 2015; Cholewiak and Collins 2003; Verrillo 1980), and so, discrimination thresholds might vary if we conduct the same experiments in an older population. In addition, the mechanical propagation of vibrations through soft tissues in the arm and hand is frequency-dependent [c.f., Manifredi et al. (2012); see also Sofia and Jones (2013)]. Thus, the number of receptors activated by a given stimulus will be frequency-dependent, as will be also the magnitude of discrimination thresholds [see also (Francisco et al. 2008)]. Finally, because vibrotactile perception also appears to depend on stimulus presentation time for short stimuli less than 1 s in duration (Berglund et al. 1967), we would also expect the magnitude of discrimination thresholds to vary slightly as a function of stimulus duration. In all of these cases, however, we would not expect the observed variations in perception across dermatomes and across temporal patterns of stimulation to change as a result of arbitrary choices in standard stimulus frequency, stimulus duration, and participant population. Future experiments of vibrotactile perception could be performed to verify these assumptions.
